# A Vulnerability Assessment for a Future HIV Outbreak Associated With Injection Drug Use in Illinois, 2017–2018

**DOI:** 10.3389/fsoc.2021.652672

**Published:** 2021-05-19

**Authors:** Cara Jane Bergo, Jennifer R. Epstein, Stacey Hoferka, Marynia Aniela Kolak, Mai T. Pho

**Affiliations:** ^1^University of Illinois at Chicago, Chicago, IL, United States; ^2^Illinois Department of Public Health, Springfield, IL, United States; ^3^University of Chicago, Chicago, IL, United States

**Keywords:** injection drug abuse, HIV, vulnerability analysis, outbreak, infectious disease, hepatitis C (HCV) infection

## Abstract

The current opioid crisis and the increase in injection drug use (IDU) have led to outbreaks of HIV in communities across the country. These outbreaks have prompted country and statewide examination into identifying factors to determine areas at risk of a future HIV outbreak. Based on methodology used in a prior nationwide county-level analysis by the US Centers for Disease Control and Prevention (CDC), we examined Illinois at the ZIP code level (*n* = 1,383). Combined acute and chronic hepatitis C virus (HCV) infection among persons <40 years of age was used as an outcome proxy measure for IDU. Local and statewide data sources were used to identify variables that are potentially predictive of high risk for HIV/HCV transmission that fell within three main groups: health outcomes, access/resources, and the social/economic/physical environment. A multivariable negative binomial regression was performed with population as an offset. The vulnerability score for each ZIP code was created using the final regression model that consisted of 11 factors, six risk factors, and five protective factors. ZIP codes identified with the highest vulnerability ranking (top 10%) were distributed across the state yet focused in the rural southern region. The most populous county, Cook County, had only one vulnerable ZIP code. This analysis reveals more areas vulnerable to future outbreaks compared to past national analyses and provides more precise indications of vulnerability at the ZIP code level. The ability to assess the risk at sub-county level allows local jurisdictions to more finely tune surveillance and preventive measures and target activities in these high-risk areas. The final model contained a mix of protective and risk factors revealing a heightened level of complexity underlying the relationship between characteristics that impact HCV risk. Following this analysis, Illinois prioritized recommendations to include increasing access to harm reduction services, specifically sterile syringe services, naloxone access, infectious disease screening and increased linkage to care for HCV and opioid use disorder.

## Introduction

HIV outbreaks related to injection drug use (IDU) in the setting of the current opioid crisis have been reported in communities across the U.S. In 2014, Scott County, Indiana identified more than 200 cases of HIV linked to the injection of prescription opioids. In subsequent years Massachusetts identified an outbreak of 116 cases with HIV that occurred among people who inject drugs (PWID)^1^ (Alpren et al., 2020), and more recently, West Virginia has experienced HIV clusters amongst the PWID communities in multiple counties^2^^,^^3^. Outbreaks of HIV have also been associated with underlying and preceding networks of hepatitis C virus (HCV) infection, which may portend future risk of HIV given the relationship with drug injection (Shepard et al., 2005; Mumtaz et al., 2015; Ramachandran et al., 2018).

The opioid crisis, which has disproportionately affected rural communities has laid bare the multiple population and environmental factors that underlay vulnerability to these infectious diseases (Keyes et al., 2014; Van Handel et al., 2016). Individual-level characteristics, such as demographic (e.g., age, race, and disability status) and socioeconomic factors (i.e., poverty status, employment, homelessness, and education) are associated with risk of HIV (Des Jarlais et al., 2020; Schalkoff et al., 2020). Low healthcare access, including infectious disease screening, prevention measures, such as syringe service programs and substance use disorder treatment can affect the spread of a disease related to injection drug use (Havens et al., 2018; Lerner and Fauci, 2019; McLuckie et al., 2019; Siegler et al., 2019). At the level of the physical environment, characteristics, such as transportation and types of housing can impact engagement in and utilization of HIV services^4^ (Aidala et al., 2016). In Illinois, fatal and nonfatal overdoses related to opioids have increased by 3% between 2018 and 2019^5^. While the major metropolitan areas have represented the majority of absolute cases, rural areas have experienced some of the highest per population rates of both overdose as well as other consequences of the opioid epidemic, such as neonatal abstinence syndrome^6^^,^^7^. HCV infection in the state has increased by 43% from 6,887 in 2006 to 9,838 in 2017, with many of the cases in individuals younger than 35 years of age and linked to injection drug use^8^.

Prior studies, including analyses performed by the Centers for Disease Control and Prevention (CDC) have examined multiple factors at the national level to identify areas at high risk of rapid HIV transmission (Rickles et al., 2018; McLuckie et al., 2019). Such work has outlined a methodological model for vulnerability analysis that has inspired states to take a finer approach in examining factors based on local data.

We offer further insight at the subcounty, ZIP code level in Illinois to what local areas are vulnerable to an HIV outbreak. Recent work in southern Illinois showed more variation at the ZIP-scale than the county-level, necessitating further investigation at the full state scale (McLuckie et al., 2019). ZIP code-level analysis provides a more granular understanding of risk within large and diverse counties in Illinois, but is also generally large enough to protect individual privacy when summarizing health records. This may allow local jurisdictions to more narrowly target specific public health interventions within these vulnerable areas in an effort to prevent future outbreaks. Additional inclusion of environmental factors will provide further contextual information to identify resource gaps that may impact outbreak risk.

## Materials and Methods

### Study Design

An ecologic study design was used to evaluate associations of HCV health outcomes and various related health outcomes, treatments, intervention, and risk factors at an environmental scale. ZIP code tabulation (ZCTA) level indicators were collected from the 2013–2017 five-year average US Census American Community Survey, Illinois state-specific indicators from the CDC, and surveillance data related to HCV, sexually transmitted diseases and fatal and non-fatal opioid-related overdose from the Illinois Department of Public Health. Data were collected for 2017 and 2018 to mirror the methodology used by CDC (Van Handel et al., 2016). Illinois' 1,383 ZCTA codes comprised the study sample. Covariates were collected and analyses conducted at the ZIP code level.

### Data

ZIP code level vulnerability to an HIV or HCV outbreak related to injection drug use was indicated by the detection incidence of HCV cases from 2017 to 2018. All confirmed and probable cases of HCV infection (chronic and acute) in individuals <40 years of age were included (Table 1). This designation was used as a proxy for acute HCV infections which are known to be underreported due to the asymptomatic and minimally symptomatic nature of most acute infections (Onofrey et al., 2015). HCV detection incidence was defined as diagnosed cases meeting a confirmed case definition for HCV infection, indicated by a positive HCV nucleic acid test or HCV antigen test. Rates were obtained from the Illinois Department of Public Health (IDPH) program and the Illinois- National Electronic Disease Surveillance System (I-NEDSS), which collects mandated infectious disease reporting from laboratories, health care providers, and other mandatory reporters.

**Table 1:** Dependent variable and indicators originally identified to be used in regression model at the ZIP code level, Illinois, 2017–2018.

	**Definition**	**Source**
**Health Outcomes**		
1. Acute and chronic HCV cases under the age of 40 years (dependent variable)	Number of confirmed HCV cases	Illinois Department of Public Health^*a*^
2.Chlamydia	Number of confirmed chlamydia cases	
3.Syphilis	Number of confirmed syphilis cases	
4.Gonorrhea	Number of confirmed gonorrhea cases	
5.Fatal and non-fatal opioid-related overdose^*^	Combined number of nonfatal and fatal opioid- related overdose	Hospital Discharge Data and Vital Statistics^*b*^^,^^*c*^
**Access/resources**		
6. Naloxone access	Pharmacy or Opioid Education and Naloxone Distribution Program utilizing the statewide Naloxone Standardized Procedure	Illinois Department of Public Health^*d*^
7. Federally Qualified Health Centers	Location of Federally Qualified Health Centers as of January 1, 2019	Health Resource and Services Administration Data Portal^*e*^
8.Drug and alcohol use disorder treatment programs	Methadone outpatient treatment clinics	Substance Abuse and Mental Health Services Administration 2018 dataset^*f*^
9.Drug and alcohol use disorder treatment programs	Buprenorphine-waivered physicians	Substance Abuse and Mental Health Services Administration 2018 dataset (see text footnote *f*)
10.Drug and alcohol use disorder treatment programs	Naltrexone providers	Substance Abuse and Mental Health Services Administration 2018 dataset (see text footnote *f*)
**Social, Economic, and Physical Environment**		
11.Black %	Percent of persons who reported they were not Hispanic or Latino and were of Black race.	United States Census Bureau. American Community Survey 2013–2017. Five-year estimates^*g*^
12.White %^*^	Percent of persons who reported they were not Hispanic or Latino and were of White race alone.	
13.Hispanic %^*^	Percent of persons who reported they were Hispanic or Latino.	
14.Over 65^*^	Percent of persons who reported they were over 65 years of age.	
15.Population 15–24^*^	Percent of persons who reported they were between 15 and 24 years of age.	
16.Disability rate^*^	Percent of persons who reported they were disabled.	
17.Poverty rate^*^	Percent of persons in poverty according to levels defined by the Census Bureau, which uses a set of income thresholds that vary by family size and composition to determine who is in poverty. If a family's total income is less that the family's threshold, then that family and every individual in it is considered in poverty.	
18. Income^*^	Mean income per person in the county; derived by dividing the total income of all people 15 years and older by the total population; modeled as log_10._	
19. Unemployment rate	Number of civilian persons unemployed and actively seeking work divided by the estimated total civilian population aged 16yrs and older.	
20. Education	Number of persons aged 25 yrs or older with less than a 12 grades education (including individuals with 12th grade, but no diploma) divided by the estimated ZIP code level population aged 25 yrs and older).	
21. Gini Index	Measure of the distribution of income across income percentiles in the population.	
22. Risky jobs^*^	Percent individuals employed in agricultural, forestry, mining, logistics/utilities, construction, and manufacturing industries.	
23. Mobile home^*^	Percent mobile home structures	
24. Vacant home	Proportion of vacant to occupied homes	
25. Rental house^*^	Percent renters	
26. Old home	Percent of persons who have lived in their home for more than 20 years.	

a *Illinois Department of Public Health. Infectious Disease Reporting. Available online at: http://www.dph.illinois.gov/topics-services/diseases-and-conditions/infectious-diseases/infectious-disease-reporting*.

b *Illinois Department of Public Health. Death Statistics. Available online at: http://www.dph.illinois.gov/data-statistics/vital-statistics/death-statistics/more-statistics*.

c *Illinois Department of Public Health. Discharge Data. Available online at: http://dph.illinois.gov/topics-services/prevention-wellness/patient-safety-quality/discharge-Data*.

d *Illinois Department of Human Services. IDHS/SUPR Initiatives in Response to the Opioid Crisis. Available online: http://www.dhs.state.il.us/page.aspx?item=105980*.

e *Health Resource and Services Administration. Data Portal. Available online at: https://data.hrsa.gov/hdw/tools/dataportal.aspx*.

f *Substance Abuse and Mental Health Services Administration. Legislation, Regulations, and Guidelines. Available online at: https://www.samhsa.gov/programs-campaigns/medication-assisted-treatment/legislation-regulations-guidelines*.

g *United States Census Bureau. Available online at: https://www.census.gov/data/datasets/2017/demo/popest/counties-total.html*.

* *Final variable in model*.

ZIP code level predictors were identified through author consensus as potentially predictive of high risk for HIV/HCV transmission and summarized across all Illinois ZCTA areas (*n* = 1383). Some indicators were collected as counts and subsequently calculated as rates, per total population between ages 15–40 years (Table 1).

Chlamydia, gonorrhea and syphilis cases were collected *via* the same mechanism as HCV. Fatal overdose rates were extracted from Illinois Vital Records: heroin deaths were assigned as any drug overdose death in which heroin (ICD-10 code T40.1) was reported as a cause of death; analgesic deaths were assigned as any drug overdose death in which prescription analgesics (methadone, synthetic narcotics, or other prescription opioids, ICD-10 codes T40.2, T40.3, T40.4) were reported as a cause of death; opioid deaths as any drug overdose death in which any opioid drug was a contributing cause of death—includes the above four ICD-10 codes as well as T40.0 (opium) and T40.6 (other/unspecific narcotics). Non-fatal opioid-related overdose rates were collected from IDPH hospital and emergency department (ED) discharge data.

To better understand access to resources throughout the state, we included several treatment and intervention variables: 1) access to a pharmacy utilizing a naloxone standing order; 2) access to clinicians or clinics that can prescribe/dispense buprenorphine, methadone, or naltrexone; 3) and access to federally qualified health centers (FQHC). These systems were included within access to resources due to their ability to serve and engage the community (Joudrey et al., 2019). Specifically, FQHC's serve medically underserved areas, provide a wide range of services, including counseling and medication-assisted treatment, and have been shown to be associated with opioid-related mortality (Haley et al., 2019; Flores et al., 2020). Access to pharmacies with a standing order for naloxone (from the IDPH registry), “drug use disorder treatment programs” (as defined by methadone outpatient treatment clinics), buprenorphine-waivered physicians with records of prescribing in the Illinois Prescription Monitoring Program, and naltrexone providers (sourced from the Substance Abuse and Mental Health Services Administration 2018 dataset), and federally qualified health centers (from study collection) were available as locations at the address level, and subsequently geocoded and converted to spatial data points. Distance from the ZIP code center to nearest facility was then calculated in QGIS software.

ZIP code level demographics were collected from the American Community Survey 2013–2017, five-year estimate (American Community Survey 2017). Covariates reflect place-based features of the Risk Environment Model that guide studies of the social determinants of HIV-related outcomes among PWID, recently extended and adapted to southern Illinois at the ZCTA scale (Rhodes, 2009; Kolak et al., 2020). Covariates were extracted to approximate varying dimensions of risk across social, economic, and physical environments as guided by review of the Risk Environment Model literature, as well as input from local and state-level taskforce meetings involving stakeholders from local health departments, emergency medical services and other first responders, community-based service organizations. and advocacy groups. Percent Whites, Blacks, and Hispanic persons were included at the ZIP code level (Keyes et al., 2014). Seniors were designated by percent of population over 64 years of age, young adults by percent of population aged 15–24 years, and percentage of persons with a disability (Keyes et al., 2014). Areas with greater proportions of seniors, young adults, and/ or persons with a disability may reflect different dimensions of neighborhood structures that can influence, interact with, and impact opioid use disorder (OUD) risk environments (Brady et al., 2017). Also included were indicators of socioeconomic status including percent of households in poverty, per capita income; percent unemployment; percent of working aged individuals without a high school diploma; and an income inequality Gini coefficient. Income was normalized for interpretability through a log transformation. “High risk” employment was proxied as the percent of individuals employed in agricultural, forestry, mining, logistics/utilities, construction, and manufacturing industries; these jobs were identified as at a greater risk of injury according to the CDC. Furthermore, variables were included that may act as physical environment indicators such as proportion of mobile homes, percent of vacant homes, percent persons who have lived in their home for more than 20 years, and percent renters (Kolak et al., 2020).

### Regression

Given the large sample size (*n* = 1383) due to using the ZIP code level approach, the number of indicators did not need to be reduced for analysis. Each variable was independently assessed for association with the outcome. To assess correlation between indicators, we developed a Spearman correlation matrix. The correlation matrix was calculated for pairwise complete observations and correlation plot was implemented with the proc corr package in SAS version 9.4 software (SAS Institute, Cary, North Carolina).

A multivariable negative binomial regression was performed for all five-digit ZIP code tabulation areas in Illinois, with ZCTA code level population as an offset. Negative binomial regression allowed for adjusting of the model variance independently of the mean compared to Poisson. Social, economic, and physical environment variables were included in the model based on a-priori hypotheses (Kolak et al., 2020). The goal was to create a parsimonious model, retaining variables only at the *p* ≤ 0.05 level. Backwards stepwise deletion was performed. Following each regression, the most non-significant variable was removed individually. This step was then repeated until all predictors were significant at *p* ≤ 0.05.

### Vulnerability Score and Ranking

The vulnerability score was developed using the final regression model. The coefficient of each significant indicator was used to compute each ZIP code's index score. This score was the predicted count value at the ZIP code level. This score was then converted to a rate by dividing twice the 2017 population since counts were from 2017 and 2018. This predicted rate value was then used to rank ZIP codes from highest to lowest, where higher scores indicated increased vulnerability. The top 10% of ZIP codes were designated as “most vulnerable” and the next 10% of ZIP codes were designated as “very vulnerable.”

## Results

All covariates were assessed for correlation. Individual rates of chlamydia, syphilis and gonorrhea were strongly correlated (>0.90). One variable was created as an overall sexually transmitted infection rate by summing values for the number of cases of chlamydia, syphilis and gonorrhea. All other predictors were not significantly correlated (<0.65) and therefore included in the model. The remaining predictors were used to model HCV infection in those under 40 years of age as a proxy for an HIV/ HCV outbreak. Vacant housing was excluded due to not being associated with HCV infection.

An association was observed between 11 covariates and HCV detection incidence [χ^2^ (1358, *N* = 1370) = 1238.71, *p* ≤ 0.001]. Of these, one was health related: (1) overdose risk (fatal and nonfatal) (estimate, 0.024; *P* ≤ 0.0001) (Table 2). Five variables reflected social characteristics across ZCTAs: (1) percentage White (estimate, 0.015; *P* ≤ 0.0001); (2) percentage Hispanic (estimate, −0.009; *P* ≤ 0.0001); (3) percentage over 65 years of age (estimate, −0.018; *P* = 0.018); (4) percentage disabled (estimate, 0.038; *P* ≤ 0.0001) and (5) percentage 15 to 24 years of age (estimate, −0.014; *P* = 0.032). Three of the indicators were economic: (1) poverty rate (estimate, 0.016; *P* = 0.008); (2) log of per capita income (estimate, −1.059; *P* = 0.002); and (3) risky jobs (estimate, −0.009, *P* = 0.031). Two variables were physical environment indicators: (1) mobile home (estimate, 0.028; *P* < 0.0001) and (2) rental housing (estimate, 0.015; *P* < 0.0001).

**Table 2:** Negative binomial regression results for final model with significant indicators.

**Parameter**	**Coefficient**	**Standard Error**	***P*-value**
Intercept	−5.94	1.62	0.0002
Overdose risk	0.024	0.005	<0.0001
Percentage White	0.015	0.002	<0.0001
Percentage Hispanic	−0.009	0.002	<0.0001
Percentage over 65 years	−0.018	0.008	0.018
Percentage population 15–24 years	−0.014	0.007	0.032
Percentage disabled	0.038	0.009	<0.0001
Percentage poverty	0.016	0.006	0.008
Log income	−1.059	0.334	0.002
Percentage in a risky job	−0.009	0.004	0.031
Percentage mobile home	0.028	0.005	<0.0001
Percentage rental housing	0.015	0.003	<0.0001

**Figure 1:**
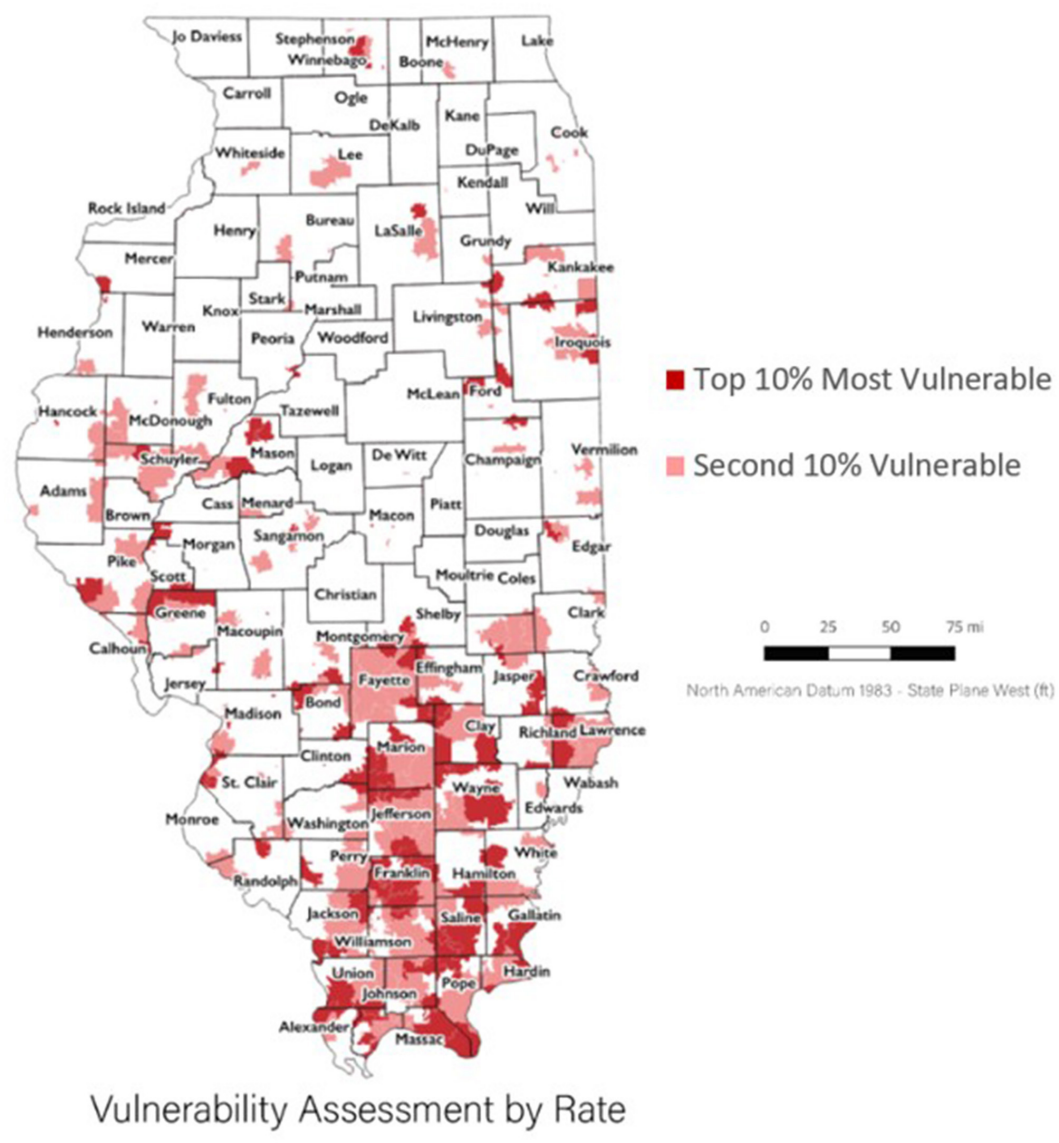
Illinois vulnerability assessment by rate.

When using our vulnerability ranking, the ZIP codes with highest vulnerability were found to be distributed across the state yet focused in the rural southern region (Figure 1). Ten counties in the more populated northern region of the state had at least one vulnerable ZIP code. Only one vulnerable ZIP code was in highly urbanized Cook County. Among the central region of the state, a group of vulnerable ZIP codes appeared around the third largest urban area in the state, the city of Peoria, in Peoria and Tazewell counties. Two rural counties in the western/ central region of the state, Mason and Greene counties, also had groupings of vulnerable ZIP codes. Two rural counties in the eastern region of the state, Iroquois and Ford counties, had a few vulnerable ZIP codes. Two counties in the southern, central region of the state were almost fully identified as vulnerable through their ZIP codes (Franklin and Saline County). Hardin counties, a southeastern rural county and the only Illinois, county identified by in the CDC analysis had two vulnerable ZIP codes.

## Discussion

We identified communities at the ZIP code level across Illinois vulnerable to an injection drug use-related HIV outbreak using statewide data sources from 2017–2018. Our finer-scaled analysis illustrated more vulnerable regions compared to the national view, providing more precise indications of vulnerability below county scale. The CDC identified one county, Hardin County, as highest risk through their assessment (Van Handel et al., 2016). This county is the least populous county in rural, southern Illinois and shares a border with Kentucky. Out of the four ZIP codes within this county, our study found that two were of the most vulnerable ZIP codes in the entire state of Illinois. We furthermore refined our understanding of HCV detection incidence in Illinois with an extended review of multiple risk factors. Areas with more Hispanic residents, seniors, college-aged students, persons employed in “high risk jobs,” and/or higher incomes were associated with decreased HCV detection incidence, whereas the remaining covariates (opioid-related overdose, areas with more White residents, persons with a disability, mobile home structures, and/or greater proportion of rental housing) were associated with increased incidence.

Illinois has often been referred to as a microcosm of the country, being representative of the national demographics related to age, race, education, and household income. While Chicago is the third most populous city in the US, nearly two-thirds of the state's 102 counties are rural. Disparities between large metropolitan and rural areas exist for a broad swath of health outcomes much as they do nationwide. While absolute cases of chronic HCV are correlated with higher population density, acute HCV incidence in the US has been increasing with a greater predominance in rural counties in association with the opioid epidemic (Zibbell et al., 2018). Consistent with these national trends and other published vulnerability analyses, ZIP codes of high risk were clustered in rural Illinois (Wesner et al., 2020). At the same time, regions of heightened vulnerability in southern Illinois identified in previous work persist in this analysis, but are put into context within the wider state's landscape (McLuckie et al., 2019). While the spread of HIV in the country is primarily driven by sexual transmission, in non-metropolitan settings injection drug use contributes to higher rates of new diagnoses as compared to urban areas (Schranz et al., 2018). These risks are also reflected in Illinois where the rate of opioid overdose increase disproportionately burdens rural counties and were identified as the significant health-related predictor in this regression model. Factors such as limited access to OUD, HIV and HCV treatment in rural settings may contribute to worse health outcomes and increased risk^9^ (Illinois Department of Public Health, 2017; McLuckie et al., 2019).

Prior analyses of Illinois public health surveillance data have demonstrated other demographic variables associated with increased risk of HCV, including white race, 25–64 age group and residence in rural counties (Jones et al., 2017). In contrast to a jurisdictional vulnerability analysis performed for South Dakota where disability was noted to be protective, in our analysis self-reported disability was associated with increased vulnerability to HCV. Given the complex association of disability with substance use disorder and that both overdose-related hospitalizations and mortality have been shown to be disproportionately represented in Medicare-disability beneficiaries, our findings are plausible and suggest that this population may benefit from focused preventive interventions (Compton et al., 2007; Glazier and Kling, 2013; Peters et al., 2018; Kuo et al., 2019).

Higher income and lower percentage in poverty were protective factors, consistent with prior vulnerability analyses (Van Handel et al., 2016; Rickles et al., 2018; Sharareh et al., 2020). Risky jobs, characterized as employment in the agricultural, forestry, mining, logistics/utilities, construction, and manufacturing industries are more prevalent in non-urban Illinois^10^. While high risk employment may be prone to work-related injury and potentially increased utilization of prescription opioids, we found the proportion of such jobs to be protective at a ZIP code level. This may be due to the higher income and employment benefits. However, in a regional analysis we previously identified southern regions of the state with a high proportion of high-risk jobs that correlated with increased opioid-related overdose prevalence, suggesting a spatially heterogeneous effect (Kolak et al., 2020). Additionally, during the timeframe of the analysis employment in the agricultural, mining and logging, manufacturing, and construction industries were stable or increasing in the state^11^. It is possible that factors, such as job stability/growth and non-payroll incentives or benefits may be mediating factors. Further research is warranted to better understand the dynamics around degrees of rurality, high risk employment, and other structural benefits not reflected in per capita income in relation to disease risk.

The percentage of mobile homes and rental housing were independently associated with HCV risk suggesting that some aspects of the physical environment may impact disease vulnerability. These factors were not included in the nationwide CDC analysis, and were not significant in the county level analysis performed in Tennessee. Of note, in the South Dakota vulnerability analysis, the percent of mobile homes was associated with lower HCV infection rate in minority dominant counties, whereas in white dominant counties, this association was not significant. Given the known relationship between housing and health, this protective effect of race would be important to explore. In studies adjusted by race, homelessness and unstable housing has been shown to be associated with sharing used syringes among people who inject drugs, and higher physical health and mental health morbidity and mortality overall (Maness and Khan, 2014; Auerswald et al., 2016; Rezaei et al., 2020). Unstable housing for renting families can result in poorer health outcomes for caregivers and their children, including increased adult depression and childhood hospitalizations, and in one study the percent of mobile homes has been inversely associated with life expectancy at the census tract level (Sandel et al., 2018; Melix et al., 2020).

The findings above reveal a greater complexity underlying the interplay between demographic, socioeconomic and environmental characteristics that impact HCV risk. In our previous work focused on a predominantly rural southern areas of the state, complex heterogeneities emerged as smaller spatial units were examined and aggregated based on common environmental typologies. In short, rural areas are not monolithic, and the ability to assess risk at the ZIP code level affords jurisdictions, the capacity to finely target relatively high-risk regions regardless of county lines and of state rankings overall.

Additionally, broadening geospatial focus beyond areas of prevalent risk to those of increased vulnerability allows for more diffuse provision of preventive public health activities. State and local health departments may engage stakeholders such as community-based organizations, first responders and other health providers, to review high-risk ZIP codes that may or may not have been areas of concern based on previous service provision. Resources may be directed accordingly to build capacity and/or engage in field activities, such as disease screening as well as harm reduction services including sterile syringe provision, sexual risk reduction, and overdose education and naloxone distribution. The attention to prevention activities on vulnerable and potentially overlooked areas provides an important opportunity to expand investigation beyond active outbreaks as detected through traditional public health data, such as passive HCV and HIV surveillance, syndromic surveillance and overdose surveillance using emergency medical services and law enforcement sources.

Our analysis has several limitations. Associations between the variables and HIV/HCV vulnerability should not be considered causal. HCV infection is known to be under reported, although it is unclear how this may vary across demographic and risk-related variables (Klevens et al., 2014). We did not have access to opioid prescribing data at the time of this analysis. Variables including opioid analgesic dosing and prescriptions per person were significant in the prior vulnerability analyses (Van Handel et al., 2016; Rickles et al., 2018; Wesner et al., 2020). Fatal overdoses in Illinois have been shown to be heterogeneous in type of opioid (prescription vs. illicit) involved, history of antecedent opioid prescribing and race (Abbasi et al., 2020). In prior analyses of the Illinois Prescription Drug Monitoring Program, rural counties in southern areas had disproportionately higher opioid prescription rates, supporting our findings of vulnerability in these ZIP codes^12^. Future analysis including this data would help elucidate the impact of opioid prescribing on HCV risk. Finally, we did not have access to HCV nor HIV treatment data. These data are incompletely available in public health surveillance datasets and are resource intensive to collect (Ly et al., 2015). Given the history of highly restrictive prior authorization criteria for direct acting antiviral medications used for HCV required by the Illinois Medicaid program, and the well-accepted strategy of treatment as prevention for HIV, incorporating this information into future models could present a more complete assessment of risk (Dieffenbach and Fauci, 2009; Granich et al., 2009; Montaner, 2011; Barua et al., 2015). Identifying vulnerable ZIP codes through our analytical approach may allow local jurisdictions to focus their limited resources on collecting treatment data at a hyper local level.

Our study updates prior, US-wide county level analysis of geospatial risk for HCV and HIV outbreak related to injection drug use with a fine-scale approach. We validated and extended previous findings to identify additional areas of vulnerability. Priority recommendations defined by the state as a result of the analysis include increasing access to harm reduction services, specifically sterile syringe services, naloxone access, infectious disease screening and increased linkage to care for HCV and opioid use disorder^13^. ZIP code level rankings allow local public health jurisdictions to more finely tune surveillance and preventive measures and target activities at a sub-county level.

## Data Availability Statement

The data analyzed in this study is subject to the following licenses/restrictions: confidential infectious disease reporting data. Requests to access these datasets should be directed to Cara Jane Bergo, cara.bergo@illinois.gov.

## Author Contributions

MP and MK helped form this paper and the overall project. SH and JE helped allow data access and explore ways to implementation, also were consistent editors of the paper. CB worked on the analyses of this paper. All authors contributed to the article and approved the submitted version.

## Conflict of Interest

The authors declare that the research was conducted in the absence of any commercial or financial relationships that could be construed as a potential conflict of interest.

## Footnotes

^1^Centers for Disease Control and Prevention (2019). Available online at: https://www.cdc.gov/mmwr/volumes/67/wr/mm6708a6.htm (accessed October, 2020).

^2^Centers for Disease Control and Prevention (2019). Available online at: https://www.cdc.gov/mmwr/volumes/68/wr/mm6810a6.htm?s_cid=mm6810a6_w (accessed November, 2020).

^3^West Virginia Department of Health and Human Resources (2019). Available online at: https://oeps.wv.gov/hiv-aids/pages/default.aspx (accessed November, 2020).

^4^Understanding Local Spatial Variation Along the Care Continuum: The Potential Impact of Transportation Vulnerability on HIV Linkage to Care and Viral Suppression in High-Poverty Areas, Atlanta, Georgia.

^5^Illinois Department of Public Health (2020). Available online at: www.dph.illinois.gov/sites/default/files/publications/semiannual-opioid-overdose-report9292020final.pdf

^6^Illinois Department of Public Health (2017). Available online at: https://www.dph.illinois.gov/sites/default/files/publications/publicationsdoil-opioid-data-report.pdf (accessed November, 2020).

^7^Illinois Department of Public Health (2019). Available online at: https://dph.illinois.gov/sites/default/files/publications/nas-annual-report-march-2019.pdf (accessed November, 2020).

^8^Illinois Department of Public Health (2018). Available online at: https://dph.illinois.gov/news/hepatitis-cases-are-increasing (accessed November 2020).

^9^Neonatal Abstinence Syndrome Advisory Committee and Illinois Department of Public Health (2019). Available online at: https://dph.illinois.gov/sites/default/files/publications/nas-annual-report-march-2019.pdf

^10^Illinois Institute for Rural Affairs. *A Snapshot of Rural Illinois. Investing in Healthy Rural Communities: Harvesting the Power of People, Place, and Partnerships*. Available online at: http://www.rwhc.com/mediasite/6-App-Chris%20Merrett_Plenary%20am.pdf

^11^Bureau of Labor Statistics. *BLS Data Finder*. Available online at: https://beta.bls.gov/dataQuery/find?fq=survey:%5Bsm%5Dands=popularity:D

^12^Opioid Prescribing in Ilinois: Examining Prescription Drug Monitoring Program Data. (2018). Available online at: https://jrsa.org/pubs/sac-digest/vol-28/il-opioidprescribing.pdf

^13^IDPH JVA Report.
